# Commentary: Basic Research in HIV Vaccinology Is Hampered by Reductionist Thinking

**DOI:** 10.3389/fimmu.2016.00266

**Published:** 2016-07-05

**Authors:** Marc H. V. Van Regenmortel

**Affiliations:** ^1^CNRS, School of Biotechnology, University of Strasbourg, Strasbourg, France

**Keywords:** HIV vaccines, reverse vaccinology, philosophy of science, bioethics of vaccine trials, reductionism

Structure-based reverse vaccinology (RV) (1) attempts to develop an HIV-1 vaccine by determining the 3-D structure of complexes of HIV-1 Env epitopes bound to broadly neutralizing monoclonal antibodies (bnMabs) (2). For more than 10 years, hundreds of investigators have tried, without success, to transform Env epitopes of known structure into effective vaccine immunogens, because they thought that epitopes that bind to affinity-matured bnMabs would also be able to induce similar antibodies when used as immunogens (3).

Using the convergence argument (4), I suggested that all these independently obtained negative results justified the conclusion that RV was unlikely to lead to a successful vaccine because it is based on invalid reductionist thinking (5). King (6) disagreed with this conclusion because it was based on inductive reasoning (i.e., generalizing from a limited body of evidence), and there was therefore no “proof” that the conclusion was correct (7). Indeed, experimental science never leads to absolute certainty, since certainties are only achieved by logical, deductive reasoning and are not derived from empirical experimentation. When Einstein declared “It makes no sense to do the same thing over and over again and expect a different result,” he did not contest that scientific conclusions are always reached by inductive inferences that may have only a reasonable probability of being correct.

My convergence argument that RV is inappropriate for developing an HIV vaccine, however, was only a back-up argument (5), since the actual reason why RV failed is that it did not have a sound theoretical basis corresponding to our current knowledge of immunological specificity and anti-HIV immune responses. For instance, RV does not take into account that the immune system is degenerate (3, 8) and that antibodies and paratopes are never monospecific for a single epitope but are always polyspecific (9) or even heterospecific (10) for a large number of epitopes. This means that a single antibody is always able to bind several epitopes, besides the one observed by X-ray crystallography of one paratope–epitope complex. Thus, there is no reason to believe that this epitope of known structure is necessarily the one that induced the antibody and could be expected to elicit bnAbs when used as vaccine immunogen.

Another theoretical misunderstanding by many proponents of RV is that they believe that when they improve the antigenic reactivity of one Env epitope with respect to a single bnMab, using molecular engineering, this amounts to “designing” a superior vaccine immunogen capable of eliciting protective antibodies (11). In so doing, they confuse antigenicity, which is a chemical property that allows a molecule to bind to an antibody, with immunogenicity, which is a biological property involving an appropriate immune system. This is typical of reductionist thinking, which assumes that biology can be reduced to chemistry and that an antigen is necessarily able to elicit the antibodies that it can react with. In fact, many factors that determine which antibodies will be produced are external to epitope–paratope recognition and originate in the immunized host. RV is also ineffective in the case of HIV, because neutralizing anti-HIV Abs are only obtained after a lengthy process of Ab affinity maturation, which is usually not the case for immune responses to other viruses. As a result, RV is not applicable to the epitopes of known structure that are recognized only by affinity-matured antibodies. Current attempts to develop vaccine immunogens from Env epitopes that do not bind germline B cell receptors or maturation intermediates present in naive individuals actually depart from the original RV strategy, which does not require the unraveling of antibody maturation pathways (11).

Another reductionist limitation of RV is that it makes use only of epitopes recognized by a limited number of bnMabs, thereby neglecting the fact that the entire surface of a protein contains a very large number of overlapping epitopes and potentially immunogenic regions (12). However, it is well-known that effective vaccine-induced antibody responses are always polyclonal and recognize a wide variety of epitopes (13).

King (6) also pointed out that HIV vaccine development and related efficacy trials in humans present numerous ethical constraints that are particularly challenging. He suggested that it may be unethical to pursue attempts to develop an HIV-1 vaccine by RV, if scarce resources could be used more effectively to combat the AIDS epidemic and its huge societal problems by other means. Such a conclusion is reinforced by the theoretical shortcomings of RV outlined above, which suggest that other vaccine approaches should rather be investigated and funded (14).

It could also be argued that the unwillingness of regulatory authorities to allow small-scale human vaccine trials, unless positive results have been obtained earlier with non-human primates (NHPs), may also be ethically questionable. It is widely accepted today that a vaccine response in NHPs (whether positive or negative) is not at all predictive of what is likely to happen in humans (15, 16). For instance, this means that a vaccine that shows no efficacy in NHPs may never be tested in humans, and thus that its possible efficacy would not be discovered because of ethical considerations. For instance, it is obvious today that the thalidomide disaster could not have been avoided if pregnant NHPs had first been tested in toxicity trials, since the drug is only teratogenic in humans (17). It should in fact be accepted that the only reliable model system for a human vaccine are human subjects (18, 19). Small-scale human trials (20, 21), using, for instance, HIV-infected individuals with temporarily interrupted ART, may be one approach that could be used to evaluate potential therapeutic HIV vaccines.

## Author Contribution

The author confirms being the sole contributor of this work and approved it for publication.

## Conflict of Interest Statement

The author declares that the research was conducted in the absence of any commercial or financial relationships that could be construed as a potential conflict of interest.
